# Medical Paraclinical Standards, Political Economy of Clinic, and Patients’ Clinical Dependency; A Critical Conversation Analysis of Clinical Counseling in South of Iran

**Published:** 2014-07

**Authors:** Ahmad Kalateh Sadati, Mohammad Taghi Iman, Kamran Bagheri Lankarani

**Affiliations:** 1 Health Policy Research Center (HPRC), Shiraz University of Medical Sciences, Shiraz, Iran;; 2 Department of Sociology and Social Planning, College of Economics, Management, and Social Sciences, Shiraz University, Shiraz, Iran;

**Keywords:** Doctor-Patient Relation, Clinic, Dependency, Political Economy

## Abstract

**Background:** Despite its benefits and importance, clinical counseling affects the patient both psychosocially and socially. Illness labeling not only leads to many problems for patient and his/her family but also it imposes high costs to health care system. Among various factors, doctor-patient relationship has an important role in the clinical counseling and its medical approach. The goal of this study is to evaluate the nature of clinical counseling based on critical approach.

**Methods: **The context of research is the second major medical training center in Shiraz, Iran. In this study, Critical Conversation Analysis was used based on the methodologies of critical theories. Among about 50 consultation meetings digitally recorded, 33 were selected for this study.

**Results: **Results show that the nature of doctor-patient relationship in these cases is based on *paternalistic* model. On the other hand, in all consultations, the important values that were legitimated with physicians were medical *paraclinical standards*. Paternalism in one hand and standardization on the other leads to dependency of patients to the clinic.

**Conclusion:** Although we can’t condone the paraclinical standards, clinical counseling and doctor-patient relationship need to reduce its dominance over counseling based on interpretation of human relations, paying attention to social and economical differences of peoples and biosocial and biocultural differences, and focusing on clinical examinations. Also, we need to accept that medicine is an *art* of interaction that can’t reduce it to instrumental and linear methods of body treatment.

## Introduction

Doctor-patient relationship is an important subject in medical sociology and anthropology of medicine. Although this topic dates back to the time of Plato,^1^ it includes new concepts and debates today. Generally, two macro-approaches can be defined as to this subject. The first approach is related to sociological Parsons’s theory^2^ and his definition of ‘sick role’ model at the doctor- patient relationship. According to Parsons’ sick role, ‘we may distinguish the role of patient as the recipient of the services of a scientifically trained professional physician’.^2^

Another approach has defined *Critical* approach that has developed in sociological and anthropological theories. This approach is based on the critical paradigm, the main debate of which is that human life consists of the hermeneutical, interpretive, and humanistic dimensions. So, human life is complex and meaningful, based on its cultural and historical context. ‘In a crucial theoretical move, the critical theorists pointed out that the ideals of objectivity, efficiency, prediction, control, and value-freedom are themselves values’.^3^ Habermas and Foucault are two prominent thinkers that have had a critical view against the modern science including medicine.^4^^,^^5^

This research has focused on the nature of clinical counseling as a main issue at medical sociology and anthropology of medicine. So, the goal of this study is to evaluate the doctor- patient interaction, with a critical approach and emphasis on the nature of this relationship in clinical counseling. The significance of this research is due to two issues.

First, Iranian society is in transition while experiencing expansive transformations in different societal arenas including the medical system.^6^ Despite these changes, costs of medical care in Iran are very high.^7^^-^^9^ This is due to expensive costs of various medical tests and paraclinical examinations. It seems that the widespread social changes, weakness of critical approach in medicine in Iran, commercialization of medicine, legal obstacles in medicine, and socio-cultural issues are related to this problem. Many of these issues can be explored in *Clinical Counseling*. When a patient is consulted, the doctor’s approach has the main effect on his/her management of disease.

On the other hand, Iranian health care policy-makers’ consideration of ethical issues reveals the second importance of the topic at stake. The Islamic Republic of Iran’s Development Plan was approved by Board of Ministers on 14^th^ of April 2012. The Plan contains about 19 instances of the concept of “ethics”, the first three principles of the document being concerned with ethics, accountability, and justice.^10^ Because one main part of clinical counseling is related to medical ethics, this research focuses on the doctor-patient relationship in the clinics with a focus on ethics. So, the research questions this study are:

-What is the fundamental construct used as the criterion for diagnosis in clinical counseling?   

-How is this construct represented in the doctor-patient interaction and how is it linguistically circulated?  

-What other constructs can be represented or conceptualized by this construct? 

## Patients and Methods

Based on the qualitative research, the sampling in this study was purposive. Selecting the sample in qualitative research, particularity in case studies, is purposeful. Since the investigator’s sampling strategy depends ultimately on the study’s aim,^11^ these cases were purposively selected due to their close representation of the objective of the present study. Accordingly, sampling depends on the goal of the research^11^ and the researcher’s interest. It means that which sample is useful for the study to fulfill the research aim. Despite a range of sampling in qualitative research, in this study, available samples were used, i.e. the samples who were willing to participate in the research since any reluctance could lead to research bias.

The study was conducted in the second major medical training center of Shiraz city, southern Iran. The researchers consulted a number of doctors of the Medical Center about the study, and the purposes of the study. 9 doctors accepted to participate in the study. Obtaining consent was done orally.  Table 1 shows the characteristics of the participants.

**Table 1:** Characteristics of the research participants

**Type of Specialty**	**Number(s) of participants**	**Gender**	**History of practice (year)**
Internist	1	Male	<5
Infectious	1	Male	>10
Endocrinologist	1	Female	<5
Surgeon	3	Male	>10 (one of them) >5 (2 of them)
Dermatologist	1	Male	>10
Anesthesiologists	1	Female	>10
Rehabilitation	1	Male	>10

About 50 consultation meetings were digitally recorded. Throughout all of the stages from recording the conversation to preparing the paper, the researchers followed the ethical codes of American Sociological Association.^12^ Thus, according to ethical considerations, the names of the doctors and patients were kept confidential. The conversations were recorded in Jan. 2014. So, among about 50 consolations recorded, 33 were selected for the case study. This selection was done based on qualitative logic named saturation. When new data doesn’t yield any new concepts, saturation happens.

Method of analysis is Critical Conversation Analysis (CCA) that is based on the conversation analysis (CA) and critical discourse analysis (CDA). Different approaches have been proposed for conversation analysis. According to Fairclough, these approaches could be divided into two categories: critical and non-critical. Critical approaches not only describe discursive practices, but also present the relations of power and ideologies, and discursive effects upon social identities, social relations, and systems of knowledge and belief.^13^ The overriding goal of critical analysis is to evaluate various dimensions of formation of a discourse in unequal relations of power. Because, according to Foucault, discourse is composed of two major elements, namely, knowledge and power, critical approaches seek to study the effects of such relations on particular social issues.  Gee has similar views indicating that critical approach beside the descriptive presentation of interactions in social relations also focuses on distribution of power and goods in interactions.^14^

As mentioned above, critical approaches to doctor-patient relationships have been studied by some researchers through mostly interdisciplinary methods.^1^^,^^15^^-^^18^ In the present study, models of Mishler^15^ and Fairclough^13^ were drawn on to evaluate a case of doctor-patient conversation from a critical perspective. Based on the model under the study, there is an interaction control exerted by the doctor in interacting with his/her patients. In this study, the aspects of this interaction were analyzed by selecting 33 cases out of 50 consultations of doctor-patient relationship that was evaluated critically with an emphasis on the notion of interaction control.

In any research project, one main topic is validation. Because this research is a qualitative study and specially was focused on the poststructuralist method, we dealt with other issues for validation. One general approach in the qualitative research is member check. We did not use this method for two reasons. First, it is related to the problems of member check that was referred to by Marvasti.^19^ The second reason is the nature of this research. Because this is based on the critical view and our approach was critical to engage physicians’ interactions; the use of member check has its own ambiguities. So, we used  new validation strategies.

As Atkinson and Housley mentioned, the postmodernist position recognizes multiple criteria for the validity of research – allowing ethical and local criteria rather than universalistic criteria to regulate the research.^20^ This means that the researcher must observe ethical issues more than other issues. On the other hand, according to Riessman, one criterion of research validity in qualitative research is trustworthiness. Riessman offered persuasiveness, correspondence, coherence, and pragmatic use as ways for approaching validation.^21^ In this research, we used persuasiveness criteria for validation. It means that during the study, the researchers tried to access the justified and reasonable analysis. In this way, research analysis in reflexive process of results and data and attention to the context of the study are the main strategies of the researchers. Also, researchers were committed to the ethical criteria of research according to Atkinson and Housley,^20^ as mentioned earlier. In this strategy, presentation of what is truth without any manipulation, and avoidance of any seduction interpretation are the main ethical strategies.

## Results

Results of the research showed some of constructs, one of which is clinic-dependency. On the other hand, asymmetrical pattern of conversation between doctor and patient is the main point of the results.

*Pattern of Conversations*

Results showed that the pattern of doctor-patient relationship was the same as *paternalistic* model in Emanuel and Emanuel^22^ view. Also, Mishler’s^15^ model and the patriarch model both postulate that there are active unequal relations in interactions, similar to the relations in this study.

On the other hand, results showed that the discourse governing the consultations was influenced by general standards and universal medical standards. The common chunks representing this discourse were: “Where are your echo test results?”, “My tests are incomplete”.  As the ultrasonography test reveals,  “Have you brought your previous test?”,  “Your previous test results are better than this one”,  “You should go for the test and return in a month’s time”,  “I’ll prescribe a re-test”,  “Repeat the re-test”,  “Just do these tests”,  “For the time being do these tests”,  “Bring the results to me later”,  “An endoscopy re-test might be needed”,  “Let me check out your blood sugar, too”,  “I’ll add a mammography, too”,  “You should get scanned in two months”  and so on. In such chunks, both doctors and patients were dependent upon test results and other clinical tests, showing two general functions: first, evaluating the illness progress though trials in which the consultations, rather than the history of the illness, were more concerned with physical examinations, and other issues about test results such as changes in numbers and figures. 

In the consultations under the study, the doctors played the role of an interviewer while the patient or the person accompanying the patient played the role of a respondent. The following is an excerpt from a case of hypothyroidism (Consultation #7):

Doctor: Hey, how old are you?

Patient: 45.

Doctor: Do you experience monthly periods?

Patient: Yea. 

Doctor: Is it regular? 

Patient:  Yea.

Doctor: Is it little or too much?

Patient: No, it’s normal.

Doctor: Isn’t it irregular: too late or too early?

Patient: No.

Doctor: Does your belly work well? Don’t you have constipation? 

Patient: No.

Doctor: Don’t you feel too tired? 

Patient: No.

Doctor: How’s your appetite? 

Patient: Good.

Doctor: Has your weight changed? 

Patient: No, it’s fine.

This exchange shows the one-sided relationship which is observed in most of consultations. The qualitative evaluation of the consultations revealed that unequal power relations gave the following specifications to the dialogues:

1. The doctors are the ones who start the dialogue.
2. The doctors are the ones who determine how much a specific topic about the patients’ disease should be discussed.
3. In cases in which patients’ opinion about their diseases does not correspond to the doctors’ views, doctors’ tend to change the subject.   
4. Doctors finish the dialogue with a mix of verbal and non-verbal interactions.  
5. Doctors explicitly or implicitly suppress the consultations.
6. The frequency of words used by doctors is higher than that of patients. 
7. Patients in all of the consultations show absolute obedience toward doctors (although patients may change their stance afterwards). 
8. Some of the consultations are finished while patients are not sufficiently persuaded.
9. In all consultations, the tone of the dialogue and the nature of words emphasize the imperative tone of doctors and the subordinate position of patients. 
10. Questions are close-ended and patients usually answer yes/no . 

*Paraclinical Standards and Patients’ Clinical Dependency*

Making patients clinic- or hospital-dependent is one of the easily observed aspects of the consultations. Such chunks as “Get a test in a month and get back for the next month”,  “Bring your test for me to check them out next month”,  “We’ll re-do the tests next month”,  “Bring the results to me”,  “Bring them along next month”,  “An ultrasonography test should be done next month and come here after that”, “Come back here in a 6 month’s time” are all related to the topic under the study and can be collectively called clinic-dependency. Generally, another function and dimension of medical discourse is to make patients clinic-dependent, which refers to establishing a discoursal atmosphere in which the patient or the one accompanying him/her are persuaded to come back while it does not seem to be necessary.

This tendency is only possible by relying on universal paraclinical standards. What doctors intend to do by making patients dependent appears to be complicated, although its output seems to be closely related to commercialization of medicine. Still, generally speaking, increasing clinic-dependency has a scientific aspect. For example, from a certain age, males should check for prostate cancer once a year and females should refer to doctors for their papanicolaou test. Moreover, for many cardiovascular diseases, etc., doctors make patients clinic-dependent in a procedure called follow-up. 

Yet, sometimes in doctor-patient interactions there is no reason for further follow-up, or personal conditions of patient such as mental, economical and social situations don’t let to do the follow-up, but doctors use universal standards to make patients clinic-dependent. As an instance, consultation #8 is about a patient with extreme thinness. In the previous consultation, the doctor had prescribed a series of tests and ultrasonography examinations. The patient referred to the doctor with test results, which did not show anything except for some blood that might have been secreted by a kidney stone. The doctor thus recommended the patient,  “There was some blood in you urine, and repeat the text just one more time; if blood is seen again, a kidney ultrasonography test would be necessary, and if not it’s OK, nothing has happened”. 

In this consultation, the patient wanted his thinness to be cured, but the doctor prescribed a kidney ultrasonography test, recommending the patient to get back in three days. In this case, the thinness problem, which was diagnosed to be genetic by the doctor, is used as a criterion for labeling the patient ill and making him clinic-dependent. This procedure led the doctor to overextend his diagnosis to other body organs, which is a conduct influenced by political economics in medicine, from the viewpoint of critical anthropology. 

Consultation #5 was a conversation between a doctor and the mother of an 11.5 year-old girl who referred to the doctor because of her daughter’s short stature. In the conversation, which lasted for 7:30 min, the doctor and the girl’s mother were engaged in an argumentative dialectic. The girl’s mother referred to the doctor from a remote village in Fars province, Iran, to find a cure for her daughter’s short stature. In the previous session, the doctor had prescribed a series of examinations to be conducted on the girl including abdominal ultrasonography. 

Bringing all of the examination results, the mother referred to the doctor’s office for the second time. The mother, who was concerned with her daughter’s short stature disorder, was now facing another problem: the activation of her daughter’s ovaries. The doctor tried to persuade the mother that through delaying precocious puberty by injection, the problem could be solved and the patient’s stature would as a result increase. Because the doctor failed to persuade the patient, finally, he appealed to power and finalized the conversation. 

Mother: So you prescribe it all. How much are the injections, expensive? Wish they don’t have side-effects to cause more trouble…. What should be done for her growth?

Doctor: **I’m not talking about her growth at all**; this will prevent her monthly periods; I’ll check out her growth myself, get it?

Mother: So after the injections she should go for sonography to see her ovaries have stopped to grow?

Doctor: **No,** **n**o, **sonography is not needed**, (yah…Um) I’ll recognize it myself; don’t worry about that stage [giggle], **when necessary I’ll prescribe sonography or examination**.

[the end of the conversation]

This consultation shows the economic problems of the patient’s family clearly. But the doctor started a new protocol, with no attention to poverty condition, based on paraclinical standards. In one chunk of this consultation, the patient’s mother said “*I’ve come a long way from a village and spent so much money *…”, which shows that she had financial concerns. Ignoring this concern, the doctor, however, introduced a new medical procedure (hormone injection) to delay the patient’s monthly periods, imposing even more costs as the result of his *medicalization* of the case. This new medical procedure would include costs of ampules, repeated ultrasonography, hormonal check-up, other blood tests, and dependence on clinic. Apart from the costs, the important question is whether such a procedure would bring about desired results (to increase the patient’s stature).

*Paraclinical Standards as a General law*

The paraclinical standards let the doctor to label *illness* to these cases and then they get dependent on clinic. So, the paraclinical standard is a “regulation”, based on which the doctor defines his/her diagnosis and treatment that leads to clinical dependency. For example, a doctor said to a patient with helicobacter pylori (who had gone through all her medical tests and procedures),

Your H.pylori infection test is negative, but the *RULE* [to get back in a month] is to stop taking antibiotics and pantoprazole two-four weeks before the procedure; well, well, then we have to repeat the procedure for H.pylori (Consultation #13).

“The rule is…” shows an unbreakable standard for the doctor. Prescribing or repeating examinations has been observed as universal standards in different consultations: repeated full blood test, mammography, abdomen ultrasonography (Consultation #15); a full procedure for H. pylori, repeated endoscopy, neurophysiological consultation, repeated consultation (Consultation #16); repeated ultrasonography, bladder strips, repeated consultation (Consultation #18); brain C.T scan, ultrasonography, repeated H. pylori test (Consultation #14). 

There is no doubt that these *RULES* imposes heavy mental pressure and costs on the patient and family and make them dependent on the clinic with these prescriptions: “You should go for the test and get back in a month”, “I’ll prescribe a re-test”, “Repeat the re-test”, “Just do these tests”, “For the time being do these tests”, “Bring the results to me later”, “An endoscopy re-test might be needed”, “Let me check out your blood sugar, too”, “I’ll add a mammography, too”, “You should get scanned in two months” and so on.

On the other hand, doctors would like the patients to follow the instructions when they recommend making a follow-up appointment in future, generally. For example, in Consultation #14, the doctor, having examined the tests within the standards, found out that the patient had not obeyed the doctor’s recommendation of a follow-up. The following is the doctor-patient reaction:  

Doctor: After, after you went on the diet, we emphasized that your stomach infection would go away.

Patient: I, I….

Doctor: You were supposed to get back [giggle].

Patient: I’m sorry I didn’t. And I didn’t even get tested for some of the tests you had kindly prescribed.  

Here, the patient expressed a kind of *confession* to justify that he did not make a follow-up appointment because it was a simple unintentional mistake, and he implicitly requested the doctor not to consider this negligence. This confession is due to dominance of the doctor’s authority over the patient’s lifeworld. The patient knows that if he doesn’t any confession, the doctor may not provide him with a suitable counseling. So, besidess paraclinical standards, use of medical authority is a complementary technique for making patient clinic- dependency, as shown in consultation #14.

## Discussion

Clinical counseling depends on the nature of doctor-patient interaction. These power relations affect the structure and specifications of medical diagnosis procedures. The present study showed that the doctor-patient interaction was in line with Mishler’s classic model,^15^ which was called paternalistic model in Emmanuel and Emmanuel.^22^ When a paternalistic approach governs the interaction, the doctor uses an interactive dialogue that is different from participatory interaction. Accordingly, the nature of power relations affects the criteria considered by the doctor. Under such circumstances, the doctor, would pay attention to the patient’s opinion and experience of his/her illness more than him/her, has to follow more valid norms (of course from medical viewpoint), and from among these norms, medical standards are the most important medical diagnosis tools.

On the other hand, response to the question “how do the physicians the make the patient dependent on their authority and clinic, according to results is simple; paraclinical standards as a more powerful instrument. When a physician says to the patient that ‘the lab data are suspected to … disease’, s(he) is making the patient dependant on the clinic or medical procedures. So, to ‘RULE OUT’ this diagnosis, the patient inevitably does the ‘FOLLOW UP’ and the first result of this reality is patients’ dependence on clinic and physician. 

Results showed the all consultations were dominated by paraclinical standards, so that the atmosphere of the consultation was so influenced by the norms of trials that led to a situation which Petryna describes this way: “WE Don’t SEE PATIENT, WE SEE DATA”. The words of one clinical trial scientist, “I don’t see patients, I see data,” reflect the recasting of the patient’s role as data to be captured, transferred, and even manipulated.^23^ In addition to the psychological and socio-economical problem of illness labeling for the patient, making clinic-dependency is the main malfunction of this procedure.

Making dependency is one part of political economy of the clinic. Generally, as Oliver points out, dependency can have two aspects: first, people’s dependency on states and particularly welfare state, which is a macro-dimension, and second, people’s dependency on specialists and professionals. Concerning the second aspect, Oliver believes that economic structures determine the roles of professionals as gatekeepers of scarce resources; legal structures determine their controlling functions as administrators of services, career structures determine their decisions about whose side they are actually, and cognitive structures determine their practice with individual people who need help. Otherwise, why would they be employed to help them?.^24^ So, we confront with a form of political economy of clinics. This political economy has two main features as follows:

1. Preserve the physician authority. Clinicians as professionals of the clinic try to control their clients (patients) with power-knowledge authority that can be called political economy of the clinic. According to researchers, political economy of the clinic is due to the context of modern imperialism^25^ or context of neoliberalism.^26^ In this context, the main result of paraclinical standards is the patients’ dependency on the clinic that reproduces the medical power on the one hand and increases the development of political economy of medicine in the other. Clinic-dependency is another part of physician-dependency. So, this dependency is reproducing the physician’s authority on the one hand, and economical gain of clinic on another. Economical gain of dependency is called commercialization of the clinic.
2. Commercialization of the clinical procedures. From the perspective of political economics, the basis for medical standardization and medicalization is medical commercialism.^26^ This commercialization forms in micro, messo and macro level of the society. This study shows one dimension of commercialization of clinics in the micro level. Recommendation of many lab data tests, different graphs, X-rays, and MRI by physicians help to develop the paraclinical industries on one hand. Due to clinic- dependency and, specially to the physician-dependency, patients must come back for better evaluation based on paraclinical standards and this is another dimension of this commercialization. To realize this commercialism, doctors would need to control the society.

So, clinic-dependency at the micro level can lead to increased physician income. However, increase in population growth, increase in diseases, especially NCDs, lifestyle changes, and increased life expectancy should also be considered. Such cultural and social conditions that make people sensitive to their health, and with the least suspect, they refer to the clinic, and doctors based on medical power with specific tools (specially paraclinical standards), make them dependent on the clinic. As a result, medical system is confronted with inequality and also increase in the costs, as Hart has mentioned.^26^

**Figure 1:**
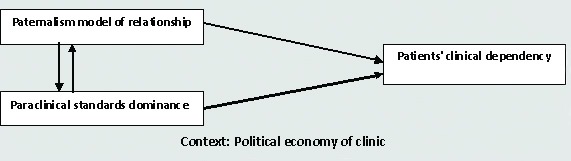
Patients’ clinical dependency in the context of clinic

According to the results, paraclinical standards have the main role in this process which is named medicalization. If medicalization means the process of considering non-medical conditions as medical problems,^27^ so it can be argued that there is a close relationship between clinic and medicalization. Govender and Peen- Kekana believe that medicalization occurs due to economic abuse so that it leads to over-medicalization for the patient, especially females.^28^ Also, Cornard believed that ‘widespread medicalization, perhaps over-medicalization, of human conditions is a trend that shows no signs of abatement’.^29^ So, physicians’ patriarchal authority based on paternalism and medical standards forms a discursive atmosphere that leads to the patients’ clinical dependency (figure 1).

According to figure 1, clinic has a powerful and rich context that reproduces its political economy. In this context, two important factors are formed including: 1. paternalistic model of doctor-patient relationship on one hand, and 2. paraclinical standards as a dominant cause on the other. And, the patient should provide profit to this context. The only way for this is clinic- dependency. So, context, model of relationship, and dominance of paraclinical standards are the three parts of the patients’ dependence on clinic. From macro level point of view, this is due to the context of modern medicine, as Foucault points out,^30^ it has provided circumstances to realize this hegemonic discourse. The results of this study confirm the above views of prominent thinkers and shows how doctor-patient relationship leads to clinicalization and medicalization based on the paternalism model and paraclinical standards. But when these results are compared with those of practical research results, new explanations are shown.

As mentioned, the analysis of 33 consultations showed that the interaction pattern in all of the cases corresponded to Mishler’s^15^ doctor-patient interaction model. In Mishler’s classical model, the consultation model was: first, a request from the doctor; second, a response from the patient; third, a post-response assessment, not always explicit, followed by a new request; and fourth, if optionally, a request for clarification or elaboration of the patient’s response. Although the classical model of Mishler has presented the nature of doctor- patient relationship well, he didn’t pay any attention to economical part of this relationship and specially clinic-dependency that was shown in this study.

Another critical approach to doctor-patient relationship is Fairclough^13^ analysis of doctor-patient relationship following Mishler analysis. The Fairclough analysis has focused on the ceremonies and politeness of the relations based on discourse analysis. He didn’t consider the economical dimension of this relationship, too; however, he has mentioned how the doctor controls the conversation. The asymmetrical relationship between doctor-patient that was revealed in this study is in the same line with Fairclough’s discussions.

Generally, this research has the same results of asymmetrical doctor-patient relation as those of Mishler,^15^ Fairclugh,^13^ Islam and Zyfur,^16^ Barry et al.,^17^ and Králová.^18^ But in this research we showed that physician power and historical authority of clinic leads to conditions called political economy of the clinic; also it was shown that between clinic-dependency and economical dimension of medicine there was a continuous covert relationship that was reproduced with paraclinical standards.

The main question is whether we can find alternative ways? Based on new approach in medicine, it can be seen that we are now confronted with new paradigmatic shift, from doctor-centeredness to patient-centeredness.^31^^,^^32^

This change suggests other models of relationship such as informative, deliberative, and interpretive model, as Emanuel and Emanuel mentioned.^20^ These models provide space for patient participation in the interaction. For example, in deliberative model, the aim of the physician-patient interaction is to help the patient determine and choose the best health-related values that can be realized in the clinical situation. To this end, the physician must delineate information on the patients’ clinical situation and then help elucidate the types of values embodied in the available options.^20^ As to the new paradigm, any consultation includes three main dimensions as Pendelton et al. mentioned; they are physical issues, psychological issues (ideas and beliefs, feelings and concerns, self regulation, narrative, expectations), and social issue. These lead to better understanding of the patient.^33^

The important point is that medical standards cannot be simply overlooked,^34^ but they need to be further adapted to cultural and economic contexts of a society and diversities of patients’ socio-economical and psychological conditions. Also, although medicalization cannot be restricted, paraclinical and medical services should be provided by a due consideration of the patient’s concerns and his/her social, economical and cultural conditions on the on hand and attention to medical prescription possible (side-)effects on the another. As Cornard argues, it is hard to imagine a world in which medicalization diminishes. Whatever the medical and social consequences, medicalization will remain a dominant approach for an increasing range of human problems. The questions remain that what is the relationship between medicalization and society’s organization; and what is the result of this relationship?.^35^ However, as Kleinman points out, medicine should be used as an art in the interaction with the patient or those accompanying the patient, rather than a mere focus on scientific data.^36^ Political economy of clinics has its ethical limitations since in clinics and also in any part of health care system “the consumers are not ‘merely consumers’- they are patients - people that are in a particular condition or state regarding a health issue”. ^37^

The main limitation of the study was the exclusion of non-verbal variables of the interaction. When a patient enters the clinic, many non-verbal interactions occur between the patient and doctor that need to be analyzed. The nature of non-verbal interaction plays an important role which can be studied in future research. So, in future studies, it is recommended that researchers should focus on video recorded data and analyze them. 

## Conclusion

This study shows that patients’ clinic dependency is the subject of clinical political economy which is the result of two elements, *Paternalism* model of doctor-patient relationship and also the dominance of paraclinical standards over the diagnosis and treatment. In this context, many issues such as humanization of relation, medical ethics, attention to physical exam and clinical standards, attention to sociocultural and economical differences between patients, and non-commercialization approach in clinic were ignored. Therefore, while we cannot condone the role of paraclinical standards in clinical counseling, its dominance needs to be reduced. Furthermore, doctor-patient relationship needs to use new approach such as informative and deliberative models.
